# MEK-inhibitor treatment reduces the induction of regulatory T cells in mice after influenza A virus infection

**DOI:** 10.3389/fimmu.2024.1360698

**Published:** 2024-06-24

**Authors:** Julia Koch-Heier, Annette B. Vogel, Yvonne Füll, Marina Ebensperger, Annika Schönsiegel, Raphael S. Zinser, Oliver Planz

**Affiliations:** ^1^ Department of Immunology, Interfaculty Institute for Cell Biology, Eberhard Karls University, Tübingen, Germany; ^2^ Atriva Therapeutics GmbH, Tübingen, Germany; ^3^ BioNTech SE, Mainz, Germany

**Keywords:** influenza A virus, Treg - regulatory T cell, MEK-inhibitor, zapnometinib, treatment strategy, immunomodulation

## Abstract

Regulatory T cells (Tregs) play a crucial and complex role in balancing the immune response to viral infection. Primarily, they serve to regulate the immune response by limiting the expression of proinflammatory cytokines, reducing inflammation in infected tissue, and limiting virus-specific T cell responses. But excessive activity of Tregs can also be detrimental and hinder the ability to effectively clear viral infection, leading to prolonged disease and potential worsening of disease severity. Not much is known about the impact of Tregs during severe influenza. In the present study, we show that CD4^+^/CD25^+^FoxP3^+^ Tregs are strongly involved in disease progression during influenza A virus (IAV) infection in mice. By comparing sublethal with lethal dose infection *in vivo*, we found that not the viral load but an increased number of CD4^+^/CD25^+^FoxP3^+^ Tregs may impair the immune response by suppressing virus specific CD8^+^ T cells and favors disease progression. Moreover, the transfer of induced Tregs into mice with mild disease symptoms had a negative and prolonged effect on disease outcome, emphasizing their importance for pathogenesis. Furthermore, treatment with MEK-inhibitors resulted in a significant reduction of induced Tregs *in vitro* and *in vivo* and positively influenced the progression of the disease. Our results demonstrate that CD4^+^/CD25^+^FoxP3^+^ Tregs are involved in the pathogenesis of severe influenza and indicate the potential of the MEK-inhibitor zapnometinib to modulate CD4^+^/CD25^+^FoxP3^+^ Tregs. Thus, making MEK-inhibitors even more promising for the treatment of severe influenza virus infections.

## Introduction

Influenza A virus (IAV) infection causes severe disease in humans and is an important topic in clinical medicine. The symptoms of the disease range from mild clinical manifestations such as fever, fatigue, sore throat, cough, headache, and limb pain to severe courses with life-threatening complications or even death (1). The immune system plays a central role in the response to IAV infections, but a delicate balance is required to fight the virus effectively without causing excessive inflammation and tissue damage (2, 3). However, in severe influenza, a dysregulated immune response leads to a massive overproduction of proinflammatory cytokines and chemokines, which determines the severity of the disease (4–6). This causes an excessive inflammatory reaction with tissue damage (7–9).

CD4^+^/CD25^+^FoxP3^+^ regulatory T cells (Tregs) play an important role in the immune response to viral infections and especially in the balance between immunity and tolerance. Their main function is to suppress effector cells such as T and B cells and maintain immune homeostasis (10). Thereby, Tregs exert an influence on various aspects of the immune response, including the regulation of cytokine dynamics, the suppression of effector T cells, the modulation of antigen-presenting cells and the modulation of inflammation (11–15). Tregs attenuate specific immune responses by controlling T helper cells and CD8^+^ T cell responses after infection (15, 16). Tregs limit the immune response after pathogen elimination, mitigate pathology and inhibit activated effector T cells (11). In some situations, excessive Treg activity can lead to immunosuppression and limit pathogen eradication or promote persistence of the pathogen, which can be detrimental. Thus, Tregs may also have a negative impact on one or more steps in the pathogenesis of viral infections. Therefore, several studies have been published in recent years investigating the role of Tregs in infectious diseases (14, 17, 18). Some studies show that Tregs protect the host from various diseases but are also involved in the severity of the disease (19–22). However, not much is known about the impact of Tregs during severe influenza. If Tregs are involved in disease progression, modulation of the Treg response during viral infection could be useful for the development of targeted therapeutic strategies.

The Raf/MEK/ERK signaling pathway is a cellular signaling cascade that plays a crucial role in regulating cell growth, proliferation, survival, and differentiation (23). The pathway is also involved in the modulation of T cell differentitaion, the activation of Tregs and is also required by influenza A and B viruses to ensure their propagation (24–26). A new approach for antiviral interventions like for COVID-19 and influenza therapy is therefore to inhibit this signaling pathway using a MEK-inhibitor that supports the antiviral effector immune response (27). Some MEK-inhibitors have been shown to have an immunomodulatory effect by reducing the expression of proinflammatory cytokines (27–29). The MEK-inhibitor zapnometinib has already been shown to have an antiviral effect against influenza A and B viruses (30). Additionally, an immunomodulatory potential to reduce proinflammatory cytokines has been observed (31). Moreover, the inhibition of MEK has the advantage of preventing resistance compared to standard antiviral drugs such as baloxavir or oseltamivir (32, 33). An influence on regulatory T cells by treatment with MEK-inhibitors is conceivable, which could improve the disease-specific immune response by helping the immune system to restore immune homeostasis.

In the present study, we therefore examined the role of Tregs in a mouse model of severe influenza and their influence on the course of the disease. To this end, we compared the viral load, the presence of various immune cells and T cell activity in mice during a mild and a severe course of the disease. In addition, we aimed to investigate the effect of MEK-inhibitors as therapeutic candidates against influenza on the regulatory T cell response *in vitro* and *in vivo*.

## Materials and methods

### Cells

Madin-Darby canine kidney cells (MDCK II, ATCC^®^, CRL-2936^TM^) were used for the determination of viral titers with foci assay and plaque assay. The cells were purchased from the American Type Culture Collection (ATCC, Manassas, Virgina, USA) and cultured in Iscove’s Modified Dulbecco’s Medium (IMDM, Thermo Fisher Scientific, Waltham, Massachusetts, USA) supplemented with 10% (v/v) fetal bovine serum (FBS, Sigma-Aldrich, St. Louis, Missouri, USA), 1% (v/v) penicillin/streptomycin (Sigma-Aldrich). Cells were maintained in a 37°C and 5% CO_2_ atmosphere. Human peripheral blood mononuclear cells (PBMCs) were isolated by density gradient centrifugation from fresh blood of healthy human volunteers (registered in the biobank of the Department of Immunology at the Eberhard Karls University of Tübingen) and used to obtain human regulatory T cells. Ethical approval was granted after review by the Ethics Board of the Medical Faculty of the Eberhard Karls University of Tübingen and the University Hospital Tübingen (887/2020BO2).

### Animals

Eight-week-old male C57BL/6 mice were obtained from the breeding facilities at the Friedrich-Löffler Institute (Federal Research Institute for Animal Health, Tuebingen, Germany) and were used for the survival study and analysis of immune cell populations. The mice had a body weight of 21 - 24 g at infection. The animals were fed with standard food which was available *ad libitum*. During this study, assessments included mortality checks, disease symptoms scores (**Supplementary Tables S1**, **S2**) and body weight. Mice were sacrificed as soon as their weight loss was above 25% compared to their initial body weight. Animal studies were approved by the Institutional Animal Care and Use Committee of Tuebingen.

Six-week-old female C57BL/6 mice (Charles River Laboratories, Sulzfeld, Germany) weighing 18 – 21 g at infection were used for the *in vivo* efficacy study of zapnometinib and standard of care treatment. The animals were fed with standard food which was available *ad libitum* Drinking water was available *ad libitum*. During this study, assessments included mortality checks, disease symptoms scores (**Supplementary Tables S3**, **S4**) and body weight. Mice were sacrificed by CO_2_ gassing 6 days post infection (p.i.). The mouse study was reviewed and approved by the Regional Council Tuebingen (IM 01/21 G).

### Virus and infection

The pandemic influenza A virus (IAV) strain A/Regensburg/D6/09 (H1N1pdm09) was obtained from the Friedrich-Löffler Institute, Federal Research Institute for Animal Health, Riems, Germany, and was further propagated on MDCK II cells in a BSL2 laboratory. Shortly, a T-75 cell culture flask containing MDCK II cells was infected with a 1:100 IAV dilution in infection medium (IMDM medium supplemented with 0.2% BSA containing penicillin and streptomycin) for 1 h at 37°C. Afterwards, 10 ml of infection medium was added to the cells and incubated for 48 h. After incubation, the cell culture supernatant was centrifuged at maximum speed for 10 min to remove cell debris. The titer of the stock was determined, and aliquots were frozen at -80°C. For infection, mice were anesthetized by intraperitoneal (i.p.) injection of ketamine (10 mg/kg) solution or by 5% isoflurane in O_2_. The mice were infected intranasally with either a low dose of 3 x 10^3^ plaque-forming units (pfu) or a high dose of 3 x 10^5^ pfu IAV in 50 μl PBS by inoculating 25 μl into each nostril. In the *in vivo* efficacy study, mice were infected with 3 x 10^5^ pfu IAV in 50 μl PBS by inoculating 25 μl into each nostril.

### Drugs

CI-1040 and zapnometinib were synthesized at ChemCon GmbH (Freiburg, Germany) and provided by Atriva Therapeutics (Tuebingen, Germany). Trametinib (GSK1120212) was purchased from ActiveBiochem (Hongkong, China). For the *in vitro* experiment, all inhibitors were prepared at a stock concentration of 10 mM in dimethyl sulfoxide (DMSO, Carl Roth GmbH, Karlsruhe, Germany) and further diluted in the respective media or buffer. For treatment of mice in the *in vivo* efficacy study, zapnometinib, oseltamivir (Hycultec GmbH, Beutelsbach, Germany) and baloxavir (Hycultec GmbH) were freshly dissolved in DMSO and then further diluted in 15% (v/v) Kolliphor EL (Sigma-Aldrich) and 80% (v/v) PBS. Each mouse received a dose of 25 mg/kg zapnometinib, 10 mg/kg oseltamivir, or 15 mg/kg baloxavir in an application volume of 100 μl of the formulation by oral gavage twice a day.

### Virus titer determination with foci assay

Virus titer was determined from lung homogenates of IAV-infected mice. Lungs were removed, weighed, and transferred into a Lysing Matrix D tube (MP Biomedicals Germany, Eschwege, Germany). Ice-cold PBS was added, the organs were shredded using the FastPrep^®^-24 (MP Biomedicals) and centrifuged for 10 min at 13,000 × g at 4°C. The supernatant was stored at -80°C until used to perform an Avicel^®^ foci assay on MDCK II cells. Briefly, MDCK II cells were seeded in 96-well plates (Greiner Bio-One, Kremsmuenster, Austria) and incubated overnight. The cells were washed with PBS and infected with serial 3-fold dilutions of the lung tissue homogenates in triplicates. After 1 h p.i., the inocula were removed and the Avicel^®^ overlay medium [1.25% Avicel^®^ (FMC Biopolymer, Hamburg, Germany), 10% Minimum Essential Media (MEM, Thermo Fisher Scientific), 0.01% DEAE-Dextran (Sigma Aldrich), 2.8% NaHCO_3_ (Merck, Darmstadt, Germany), 1% Penicillin/Streptomycin (Sigma Aldrich), 0.2% BSA (Carl Roth, Karlsruhe, Germany), 1% L- Glutamine (Sigma Aldrich)] was added for 24 h. The cells were fixed and permeabilized with PBS containing 4% Roti^®^ Histofix (Carl Roth). Afterwards, cells were immunostained with mouse anti-IAV nucleoprotein monoclonal antibody (Bio-Rad, California, USA) followed by goat anti-mouse IgG-HRP (Jackson ImmunoResearch Laboratories, Pennsylvania, USA). After washing, True Blue™ peroxidase substrate (SeraCare Life Science, Milford, USA) was added to detect foci, which were counted. The virus titer is given as the logarithm to the base 10 of the mean value. The detection limit for this test was<1.7 log_10_ foci forming units (ffu)/ml.

### Virus titer determination with plaque assay

To determine the reduction of viral titers in the *in vivo* efficacy study of zapnometinib and standard of care treatment, mice were sacrificed by CO_2_ gassing on day 6 p.i. Lung homogenates in PBS were prepared as described above and serially diluted (5-fold dilutions starting at a 1:5 initial dilution) in IMDM medium supplemented with 0.2% BSA containing penicillin and streptomycin. The serial dilutions were added to MDCK II cells (seeded in 12-well plates (Greiner) 24 h before) and incubated for 1 h at 37°C. Subsequently, the inoculum was removed, the cells were overlaid with Avicel^®^ overlay medium and incubated for 48 h at 37°C with 5% CO_2_. Afterwards, the Avicel^®^ overlay medium was removed, cells were washed twice with PBS, and fixed with 4% Roti^®^ Histofix in PBS for 30 min at 4°C before staining with 1% crystal violet. The plaques were counted, and the results were expressed as pfu/ml with a detection limit of 17 pfu/ml.

### Flow cytometry analysis and antibodies

For flow cytometry analysis of immune cells of the lung or thymus, mice were infected with either the low or the high dose of IAV and sacrificed on day 3, 6, or 9 p.i. by CO_2_ gassing. Organs were removed, minced, and lungs were digested in RPMI 1640 medium (Thermo Fisher) together with 0.125% collagenase II (Roche, Basel, Switzerland) for 30 min at 37°C and 5% CO_2_. Then, the digested fractions were passed through a 70 µm BD cell strainer and washed three times. Cell pellets were resuspended in fluorescence-activated cell sorting (FACS) buffer (PBS containing 2 mM ethylenediaminetetraacetic acid (EDTA) and 2% (v/v) FBS) and stained with the required fluorochrome-conjugated antibodies at a dilution of 1:100 in FACS buffer for 30 min at 4°C. After incubation, cells were washed with FACS buffer, fixed, and analyzed by flow cytometry using FACS Calibur (BD Bioscience, Heidelberg, Germany). The following monoclonal anti-mouse fluorochrome conjugated antibodies were purchased from BD Bioscience and were used for the staining of immune cells: CD3 (CD3ϵ chain), CD4 (GK 1.5, H129.12), CD8a (Ly-2, 53–6.7.), CD25 (PC61), CD11b (M1/70), CD11c (HL3). The anti-mouse antibodies against CD244.2 (eBio244F4), NK1.1 (PK136), Ly6G (RB6–8C5) and foxp3 (FJK-16s) were purchased from eBioscience (NatuTec, Frankfurt, Germany). The following gating strategy was employed: First, lymphocytes were gated using an SSC-A/FSC-A lymphocyte gate, followed by FSC-A/FSC-H duplet exclusion (single cell gate). Subsequently the gate for CD3^+^ T cells was applied, followed by gating for CD4^+^ and CD8^+^ T cells. For the CD4^+^/CD25^+^FoxP3^+^ Tregs, the double positive CD25^+^FoxP3^+^ cell population was gated from CD4^+^T cells. The antibodies CD11b (M1/70), CD11c (HL3), CD244.2 (eBio244F4), NK1.1 (PK136), and Ly6G (RB6–8C5) were utilized for the detection of DCs, macrophages, neutrophils, NK and NKT cells. The detection of the mouse Tregs was performed with the mouse Regulatory T cell staining kit (Thermo Fisher Scientific) and the mouse Treg Detection kit (Miltenyi Biotec, Bergisch Gladbach, Germany) using CD4-VioBlue^®^, CD25-APC, and anti-FoxP3-PE according to manufacturer’s protocol. The cells were analyzed by FACS Canto II (BD Bioscience) with the Diva software V.6.1.2 and for data analysis, FlowJo software (version 10, TreeStar Inc., USA) was used. The gating strategy of the mouse Tregs was carried out in several sequential steps. First, the lymphocytes were gated with a SSC-A/FSC-A lymphocyte gate, followed by the FSC-A/FSC-H duplet exclusion (single cell gate). Subsequently, dead cells were excluded with Aqua-Live Dead/FSC-A (live cell gate). The CD4^+^/CD25^+^ T cells were then gated from the living cells using CD25-APC/CD4- VioBlue^®^. This double-positive population was then used to gate the CD4^+^/CD25^+^FoxP3^+^ cell population. For the detection of the Tregs isolated from human blood donors, the Treg Detection Kit with following fluorochrome conjugated antibodies was purchased from Miltenyi Biotec: CD45-VioBlue^®^, CD4-VioGreen^®^, CD25-VioBright^®^, CD127-PE, anti-FoxP3-Vio667^®^. The antibody staining was performed according to manufacturer´s protocol and the cells were analyzed by FACS Canto II (BD Bioscience) with the Diva software V.6.1.2. For data analysis, FlowJo software (version 10, TreeStar Inc., USA) was used. The gating strategy of the human Tregs was also carried out in several consecutive steps. First, the lymphocytes were delimited with an SSC-A/FSC-A lymphocyte gate, followed by FSC-A/FSC-H duplet exclusion (single cell gate) and dead cell exclusion (live cell gate). The enriched CD4^+^ T cells were then gated by FSC-A/CD4-VioGreen^®^, followed by the CD45^+^CD25^+^ double-positive population, from which the gate for the FoxP3^+^ cell population finally followed.

### Cytokine quantification

For cytokine staining, a Th1/Th2 panel was utilized using the Bio-plex 200 system (Bio-Rad, Munich, Germany). Briefly, IAV infected mice were sacrificed on day 6 p.i. and serum samples of the mice were harvested. Therefore, blood was collected in serum sample tubes (Sarstedt, Nümbrecht, Germany) and centrifuged at 13,000 ×g for 5 min. These serum samples were added to the plate of the Bio-plex mouse cytokine Th1/Th2 kit and assayed for cytokine expression according to the manufacturer’s protocol. The measured cytokine concentration was then expressed in pg/ml by the instrument’s own software.

### *In vitro* cytotoxicity assay

The *in vitro* cytotoxicity assay was used to detect cytotoxic cells (effector cells) in the mouse. In this method, radioactively labeled chromium-51 (^51^Cr) target cells are loaded with a specific IAV peptide. Specific lysis of the target cells caused by the effector cells and the radioactive amounts released as a result can be measured. For this purpose, lymphocytes were isolated from the lungs of IAV-infected mice by density gradient centrifugation and served as effector cells. These effector cells were serially titrated at 3-fold dilutions in MEM medium in a 96-well plate. MHC-I type H-2D expressing MC57 cells (Murine fibrosarcoma cell line, H-2b from a C57BL/6 mouse) served as target cells and were radiolabeled with 0.2 m Ci ^51^Cr for 1 h at 37°C and loaded with 10 μg/ml of the immunodominant IAV peptide (NP)_366-374_ with the amino acid sequence ASNENVEIM. Subsequently, these target cells were co-incubated with the effector cells at effector to target cell ratios of 100:1, 30:1, or 10:1. Target cells in medium served as a control to measure spontaneous lysis (release of control) whereas target cells with HCl (Honeywell, Charlotte, North Carolina, USA) served as a control to measure the maximal target cell lysis and associated ^51^Cr release (maximum release). After 6 h incubation, supernatants were collected and measured for the presence of released ^51^Cr using the MicroBeta Counter 1750 (Wallac, Perkin Elmer, Waltham, Massachusetts, USA). The percentage of specific lysis was calculated according to the following formula [which was previously described in (34)]:


$$\text{\% specific lysis =}\frac{\text{release of effector cells - release of control}}{\text{maximum release - release of control}}\cdot 100$$

### Adoptive Treg transfer

Ten donor mice were infected intranasally with a high dose of 3 x 10^5^ pfu IAV. In parallel, mice were previously chipped with an E-Mitter to monitor gross motor activity and temperature using the VitalView hardware and software system (Mini Mitter, USA). Gross motor activity and temperature were recorded every 5 minutes. A daily average is displayed for a period of 24 days. Mice were infected intranasally with a low infection dose of 3 x 10^3^ pfu IAV. Three of these mice served as acceptor mice. At day 4 p.i., donor mice were sacrificed, splenic regulatory T cells were isolated using the mouse CD4^+^/CD25^+^ Regulatory T Cell Isolation Kit (Miltenyi Biotec). 1 x 10^6^ isolated Tregs were subsequently applied intravenously (i.v.) to each of the three acceptor mice. In parallel, three chipped mice each received 0.6 mg/kg rapamycin (Merck) in PBS i.p. on day 4, 5, and 6 p.i. The remaining three mice served as controls without treatment. The health status of all chipped mice was monitored from the day of infection. Mice were weighed daily, and body temperature and disease symptoms score (DSS) were monitored until day 24 p.i.

### *In vitro* efficiency of MEK-inhibition on regulatory T cell activation

To study the effect of MEK-inhibition on the induction of Tregs, regulatory T cells were isolated from human PBMCs using the CD4^+^CD25^+^CD127^dim/-^ Regulatory T cell Isolation Kit II and the Mini MACS Separator (Miltenyi Biotec) according to the manufacturer’s instructions. Briefly, the isolation of Tregs was performed in a two-step procedure. First, a cocktail of biotinylated antibodies and anti-biotin MicroBeads was used for depletion of non-CD4^+^ and CD127^high^- cells. In the second step, the flow-through fraction of pre-enriched CD4^+^CD127^dim/-^ T cells were labeled with CD25 MicroBeads for subsequent positive selection of CD4^+^CD25^+^CD127^dim/-^ regulatory T cells. The isolated cells were resuspended in RPMI medium (RPMI 1640, Gibco) containing 10% serum, 1% penicillin/streptomycin, and 1% L-Glutamine with or without Interleukin-2 (R&D Systems, Minneapolis, Minnesota, USA), which served as a booster for the expansion of the cells to greater extend and were incubated for 20 min in the presence or absence of MEK inhibitors. Afterwards, cells were stimulated with anti-CD3/CD28-coated MACSiBeads (Miltenyi Biotec) for 36 h and measured by flow cytometry using FACS Canto II (BD Bioscience).

### *In vivo* efficacy of zapnometinib versus standard of care

To examine the effect of zapnometinib and standard of care treatment on IAV infection, 20 female mice (5 per treatment group) were infected intranasally with 3 x 10^5^ pfu in 50 μl on day 0. Another 5 female mice served as uninfected control (Mock). At 24 h p.i., the mice were treated twice daily with either solvent control (5% (v/v) DMSO, 15% Kolliphor EL and 80% PBS) or 25 mg/kg zapnometinib, 10 mg/kg oseltamivir or baloxavir at a dosage of 15 mg/kg body weight, respectively. The compounds were administered via oral gavage with an application volume of 0.1 ml. The bidaily treatment was continued until day 3 p.i. and the study was terminated 6 days p.i. Lungs were harvested, dissociated into single-cell suspension using the Lung Dissociation Kit and the gentleMACS Dissociator (Miltenyi Biotec, program 37C_m_LDK_1) according to manufacturer´s protocol. The cell suspension was divided into two parts, one used to isolate Tregs using the mouse CD4^+^/CD25^+^ Regulatory T Cell Isolation Kit (Miltenyi Biotec) according to the manufacturer’s instructions and the other part was stored at -80°C until used for the determination of the virus titer by plaque assay.

### Statistical analysis

Unless otherwise noted, data were collected in MS Excel, and statistical analyses were performed using GraphPad Prism software version 9 (GraphPad Software Inc.). Data from at least three independent experiments are presented as the mean ± standard deviation (SD). Sample size (n) is indicated in the figure legends. Statistical details for each experiment are described in the corresponding figure legends. Differences in viral titer and cell populations between low and high dose were analyzed by two-way ANOVA. Differences between the treatment groups were analyzed by one-way ANOVA. Significance in percent survival was analyzed using the log-rank test (Mantel-Cox). P values< 0.05 were considered statistically significant.

## Results

### Low and high dose of influenza A virus infection revealed differences in severity of disease but not in viral load

**Figure 1:**
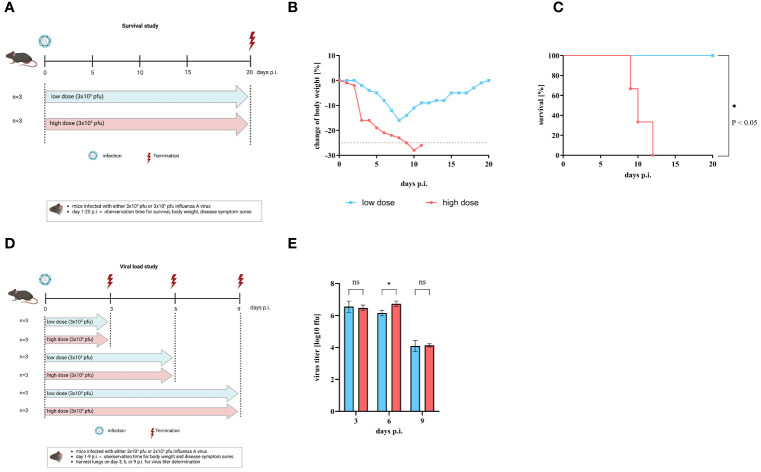
Comparison of mice after infection with low or high infection dose of influenza A virus. C57BL/6 mice were infected with either low dose (3 x 10^3^ pfu, blue line) or high infection dose (3 x10^5^ pfu, red line) of influenza A virus strain A/Regensburg/D6/09. **(A)** Scheme of the mouse survival study. Change of body weight, disease symptoms score, and survival were observed until day 20 p.i (n = 3). Figure created with BioRender.com. **(B)** Mean body weight change of infected mice (n = 3). Mice that lost over 25% of their initial body weight (grey dotted line) were sacrificed according to animal protection law. **(C)** Percentage of survival. Significant differences were analyzed by log-rank (Mantel-Cox) test (*p< 0.05). **(D)** Scheme of the mouse study for virus titer assessments. Figure was created with BioRender.com. **(E)** Virus titers of the lung are given as log10 in ffu per 1 ml organ-homogenate (n=3). Values are delineated as mean ± SD. The detection limit of this test was <1.7 log10 ffu/ml. Significance was analyzed by two-way ANOVA test (* p< 0.05). ns means not significant.

To investigate the influence of the amount of influenza A virus (IAV) used for infection on the severity of disease, C57BL/6 mice were intranasally infected with either a low dose (3 x 10^3^ pfu) or a high dose (3 x 10^5^ pfu) of IAV strain A/Regensburg/D6/09 (H1N1pdm09). Body weight, disease symptoms score (DSS), and survival were monitored for 20 days (**Figure 1A**). According to animal protection law, mice were sacrificed when body weight loss was > 25%. All mice started to lose weight 3 days p.i. Mice infected with the low dose of IAV reached a maximum body weight loss of 16% on day 8 p.i. Thereafter, the mice regained weight and reached to their initial body weight at day 20 p.i. (**Figure 1B** blue squares). In contrast, mice infected with the high dose showed a more rapid and severe decrease in body weight from day 3 p.i. (**Figure 1B** red circles). Finally, the manifestation of severe symptoms, including weight loss, culminated in mortality by day 12 p.i. at the high infection dose, while all mice infected with the low dose survived (**Figure 1C**). We then investigated whether differences in viral load in the lungs contributed to variations in disease progression. For this purpose, mice were infected with either the low or high dose of IAV and viral titers in the lungs were determined on day 3, 6, or 9 p.i. (**Figure 1D**). We found similar viral titers in the lungs of both doses on day 3 p.i. (low dose: 6.55 ± 0.35 ffu/ml, high dose: 6.47 ± 0.18 ffu/ml), on day 6 p.i. (low dose: 6.15 ± 0.18 ffu/ml, high dose: 6.73 ± 0.17 ffu/ml), and on day 9 p.i. (low dose: 4.09 ± 0.35 ffu/ml, high dose: 4.13 ± 0.10 ffu/ml) (**Figure 1E**). Thus, by using the two different doses for IAV infection, we were able to provide a sublethal and lethal influenza mouse model.

### Rapid increase of T cell response and regulatory T cells after high dose infection

**Figure 2:**
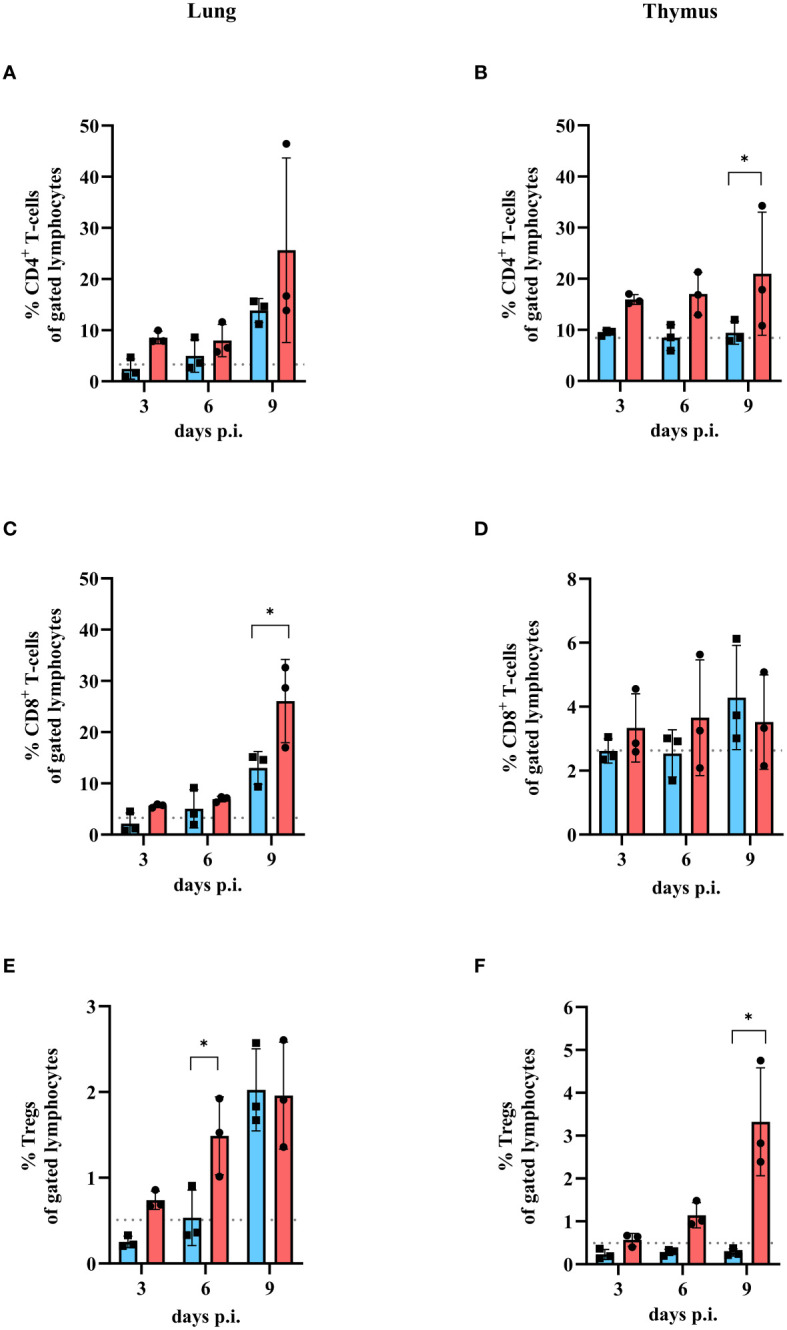
Amount of CD4^+^ T cells, CD8^+^ T cells and Tregs in lung and thymus of mice infected with either low or high dose of influenza A virus. Mice were infected with either a low dose (blue bars) or high dose (red bars) of IAV. Uninfected mice were used as control (grey dashed line). Lungs and thymi were harvested and analyzed by flow cytometry for the presence of different T cell populations at day 3, 6 or 9 compared to uninfected control mice (grey dashed line). **(A)** CD4^+^ T cell, **(C)** CD8^+^ T cell and **(E)** Treg population in the lung and **(B)** CD4^+^ T cell, **(D)** CD8^+^ T cell and **(F)** Tregs in the thymus were analyzed. Data presented as mean ± SD (n = 3). Two-way ANOVA revealed significant differences between the two groups at the same time point (black * with brackets, p< 0.05).

The fact that the virus titer was comparable after low and high dose IAV infection indicates that the viral load alone is not responsible for the development of severe clinical disease in mice. Therefore, the T cell response in mice after infection with either a low or a high dose of IAV was examined. The frequencies (in %) of CD4^+^ T cells, CD8^+^ T cells, and CD4^+^/CD25^+^FoxP3^+^ regulatory T cells (Tregs) in the lung and thymus were determined on day 3, 6 and 9 post infection (**Figure 2**). Only on day 9 p.i. significantly higher levels of CD4^+^ T cells (low dose: 13.84 ± 2.34%; high dose: 25.65 ± 18.04%, **Figure 2A**) and CD8^+^ T cells (low dose: 13.04 ± 3.21%; high dose: 26.07 ± 8.13%, **Figure 2C**) were detected in the lung for both low dose (blue bars) and high dose (red bars) infection of IAV compared to uninfected controls (CD4^+^ T cells: 3.31 ± 0.57%; CD8^+^ T cells: 3.28 ± 0.59%, grey dots). At earlier time points (day 3 and 6 p.i.), the amount of CD4^+^ T cells (day 3: 8.58 ± 1.22%; day 6: 7.98 ± 3.15%, **Figure 2A**) and the amount of CD8^+^ T cells (day 3: 5.63 ± 0.36%, day 6: 6.94 ± 0.55%, **Figure 2C**) in the lungs of mice infected with the high dose was slightly increased compared to uninfected controls (dashed line), whereas the amount of CD4^+^ T cells (day 3: 2.42 ± 1.98%; day 6: 4.96 ± 3.19%, **Figure 2A**) and the amount of CD8^+^ T cells (day 3: 2.16 ± 2.06%, day 6: 5.05 ± 3.72%, **Figure 2C**) in the lungs of mice infected with the low dose was comparable to uninfected controls (**Figures 2A, C**). Significantly higher amounts of regulatory T cells in the lungs of mice infected with high dose of IAV were detected at day 6 p.i. (1.49 ± 0.46%) and day 9 p.i. (1.96 ± 0.62%, **Figure 2E**, red bars) compared to uninfected control. After a low infection dose, a significant increase in Tregs could be detected only on day 9 p.i. (2.02 ± 0.48%, **Figure 2E**, blue bar) compared to uninfected controls (0.51 ± 0.17%, **Figure 2E**, grey dots). In the thymus of low dose infected mice, no expansion of CD4^+^ T cells was observed (day 3: 9.45 ± 0.54%; day 6: 8.49 ± 2.52%; day 9: 9.42 ± 2.23%, **Figure 2B**, blue bars) compared to uninfected control (8.44 ± 1.08%, **Figure 2B**, grey dots). In contrast, a slight increase in CD4^+^ T cells after high infection dose was shown for all timepoints (day 3: 15.93 ± 0.94%; day 6: 17.04 ± 4.18%; day 9: 20.99 ± 12.04%, **Figure 2B**, red bars). No significant increase in the amount of CD8^+^ T cells was observed at any of the time points or infection doses (day 3: 2.62 ± 0.38% (low dose); 3.34 ± 1.07% (high dose); day 6: 2.54 ± 0.74% (low dose); 3.65 ± 1.81% (high dose); day 9: 4.29 ± 1.63% (low dose); 3.52 ± 1.47% (high dose), **Figure 2D**) compared to uninfected controls. In contrast, the amount of Tregs in the thymus was altered after high dose (day 3: 0.57 ± 0.15%; day 6: 1.14 ± 0.29%; day 9: 3.32 ± 1.26%) but not after low infection dose (day 3: 0.23 ± 0.12%; day 6: 0.28 ± 0.07%; day 9: 0.27 ± 0.09%) compared to uninfected control (0.49 ± 0.15%). Significant differences were observed 6 and 9 days p.i. (**Figure 2F**).

### Different Th1 orTh2 T cell responses after influenza A virus infection

**Figure 3:**
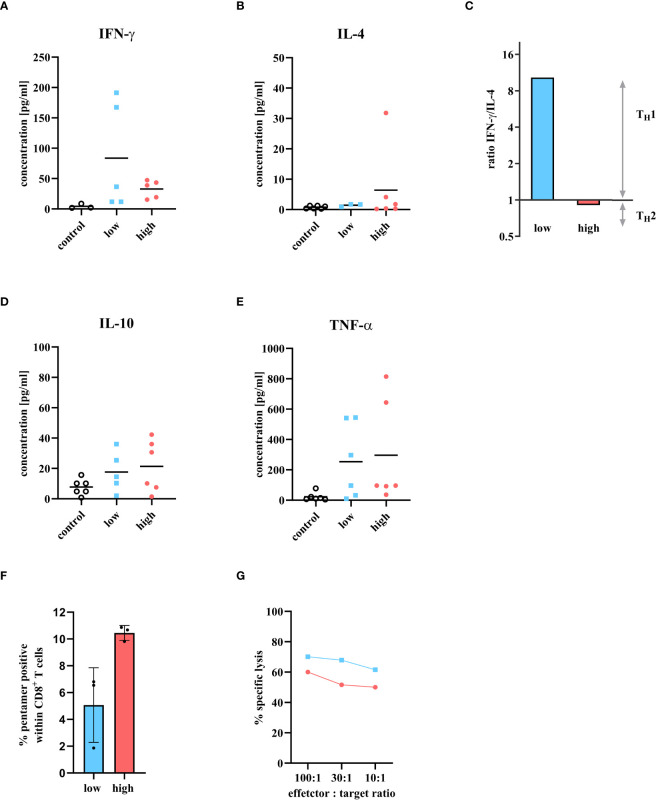
Cytokine detection of T cell-specific immune response in serum at day 6 p.i. and presence of cytotoxic T cell activity. Mice were infected with either a low or high dose of IAV and T cell specific cytokines were detected in serum 6 days p.i. The levels of **(A)** IFN-γ, **(B)** IL-4, **(D)** IL-10, and **(E)** TNF were measured using the Bio-plex 200 system and compared to uninfected control mice. The groups consisted of three mice each. The samples were measured twice independently. **(C)** The ratio of IFN-γ/IL-4 is used to determine the Th1/Th2 profile. A ratio above 1 means there is a predominance for Th1 response, and a ratio between 0 and 1 stands for a Th2 immune response. **(F)** Influenza A virus specific CD8^+^ T cells in the lungs 6 days p.i. with low dose (blue bar) or high dose (red bar) analyzed by flow cytometry. Percentage was determined by fluorescent pentamers in the CD8^+^ T cells. Values shown as mean ± SD (n = 3) **(G)** *In vitro* cytotoxicity assay using IAV specific CD8^+^ lymphocytes from the lungs of mice infected with either low dose (blue squares) or high dose of IAV (red circles). Target cells were labeled with ^51^Cr and loaded with the immunodominant influenza peptide NP_366-374_. The NP specificity of the CD8^+^ T cells were determined by the ^51^Cr release of lysed target cells. Values reflect the mean of two measurements from a cell pool of two animals each.

To investigate the quality of the CD4^+^ T cell response in more detail, interferon-gamma (IFN-γ) and interleukin 4 (IL-4) cytokine response was analyzed on day 6 p.i. in the serum of mice infected with either a low dose or a high dose of IAV. Then, the IFN-γ/IL-4 ratio was determined to investigate whether the cells showed a Th1 or Th2 CD4^+^ T cell origin. A ratio of 1 and above indicates a Th1 response, while a ratio below 1 signals a Th2 response (**Figure 3**). The serum IFN-γ concentration of infected animals was increased after both infection doses compared to control (4.27 pg/ml). A 2.5-fold higher concentration of IFN-γ was detected in mice infected with the low dose (87.75 pg/ml) than in animals infected with the high infection dose (32.88 pg/ml, **Figure 3A**). In contrast, higher concentrations of IL-4 were detected in the serum of mice infected with the high dose (6.41 pg/ml) than in mice infected with the low dose (1.44 pg/ml, **Figure 3B**). The ratio between IFN-γ and IL-4 at day 6 p.i. demonstrated that the low dose tended to result in a Th1 cytokine profile (ratio >1) while the high dose tended towards a Th2 cytokine expression (**Figure 3C**). In addition to IFN-γ and IL-4, the amount of IL-10 and tumor necrosis factor alpha (TNF) as representatives of a proinflammatory response were investigated. An increase of both cytokines was observed for both infection doses compared to the control (IL-10: 7.75 pg/ml; TNF: 22.89 pg/ml). IL-10 (low dose: 17.65 pg/ml; high dose: 21.35 pg/ml) and TNF (low dose: 253.44 pg/ml; high dose: 296.59 pg/ml), showed comparable concentrations in the serum of mice infected with the low and high infection dose (**Figures 3D, E**).

### Presence of cytotoxic T cell activity in the lungs after influenza A virus infection

Next to the CD4^+^ T cell response, the cytotoxic T cell response was scrutinized in more detail. The proportion of IAV specific CD8^+^ T cells was quantified. Mice were infected with either low or high dose of IAV and sacrificed on day 6 p.i. A cell suspension was prepared from the lung cells and analyzed by FACS for the presence of CD8^+^ T cells that were specific for the immunodominant IAV peptide NP_366-374_. Although the frequency of CD8^+^ T cells was similar between the low and high dose (**Figure 2C**), more pentamer-positive CD8^+^ T cells were identified after high dose infection (10.45 ± 0.55%) within, implying more virus specific CD8^+^ T cells after high dose infection (**Figure 3F**). The question now arose whether the higher amount of virus specific CD8^+^ T cells would also correlate with higher cytotoxicity of these CD8^+^ T cells. Therefore, an *in vitro* cytotoxicity assay was performed. Mice were infected with either a low or high dose of IAV and sacrificed on day 6 p.i. Lung lymphocytes were isolated and co-incubated with target cells labeled with the immunodominant IAV peptide NP_366-374_ to determine the specific lysis of cytotoxic T cells. After infection with the low dose, a 10% higher cytotoxic T cell activity was observed compared to the high dose (**Figure 3G**).

### Adoptive transfer of CD4^+^/CD25^+^FoxP3^+^ Tregs into mice infected with a low dose of influenza A virus led to severe disease symptoms

**Figure 4:**
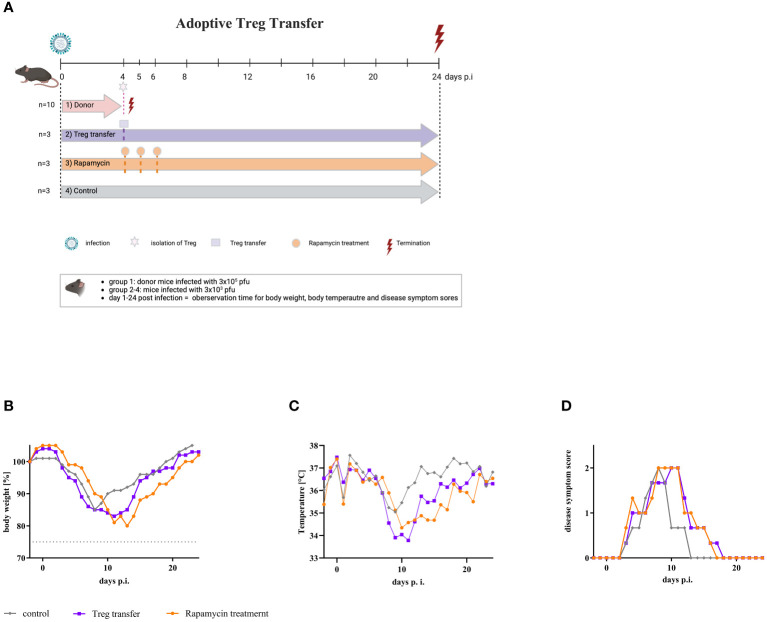
Adoptive transfer of Tregs into mice infected with a low dose of IAV resulted in severe disease symptoms. **(A)** Scheme of adoptive transfer study. Nine mice were infected with the low dose of IAV (3 x 10^3^ pfu) (purple, orange, and grey arrow). In parallel, 10 donor mice were infected with the high dose (3 x 10^5^ pfu) of IAV (red arrow). On day 4 p.i., these mice were sacrificed, and Tregs were isolated from the spleen. 1 x 10^6^ cells each of this Treg fraction were administered intravenously to all infected mice of group 2 (purple) (n = 3). In parallel, rapamycin was administered intraperitoneally to all infected mice of group 3 (orange) (n = 3). This treatment was repeated on day 5 and day 6 p.i. Animals in group 4 (grey) served as infected, untreated controls (n = 3). Observation parameters included **(B)** change of body weight, **(C)** temperature as the mean of the day, and **(D)** disease symptom scores.

Due to the different effector immune response in the lungs of mice infected with either low or high dose of IAV, one might speculate that CD4^+^/CD25^+^FoxP3^+^ Tregs inhibit the T cell effector response after infection with a high dose of IAV. Therefore, to better understand the impact of CD4^+^/CD25^+^FoxP3^+^ Tregs on influenza disease progression, Tregs were induced by high dose IAV infection. An adoptive transfer was performed by transferring these Tregs from donor mice into recipient mice, which were infected with the low dose of IAV. Furthermore, additional infected mice were administered rapamycin three times starting on day 4 p.i. Rapamycin itself is known to expand Tregs *in vitro* and *in vivo* (35). Mice infected with low dose of IAV served as controls. **Figure 4A** shows the scheme of the experimental procedure. Body weight, temperature and severity of disease were monitored daily. All infected mice lost weight from day 3 p.i. onward. Mice in the control group reduced their weight to a maximum of 85% by day 8 p.i., then regained weight and returned to initial weight by day 19 p.i. (**Figure 4B**, gray line). Mice treated three times with rapamycin began to lose weight at the same time and reduced their weight to 80% by day 13 p.i. (**Figure 4B**, orange line). These mice also regained weight and reached their initial weight at day 22 p.i. Mice that received a single transfer of Tregs 4 days p.i. also began to lose weight at day 3 p.i. and reached their minimal weight of 83% at day 12 p.i. (**Figure 4B**, purple line). By day 21 p.i., they also regained their initial body weight. As mice have a loss in body temperature when mobility and activity drop down, body temperature was measured. The body temperature of the mice also started to decrease from day 3 p.i. onwards. In mice of the control group, body temperature decreased to a minimum of 34.7°C by day 8 p.i. In comparison, the body temperature of rapamycin-treated mice decreased to 33.7°C by day 11 p.i., and the body temperature of mice receiving Treg transfer decreased to 33.4°C by day 9 p.i. (**Figure 4C**). In addition, disease symptoms were monitored (**Figure 4D**). In all mice, the first disease symptoms appeared on day 4 p.i., a time when rapamycin treatment or adaptive transfer of Tregs began. Disease symptoms in mice from the control group increased by day 8 p.i., reaching a maximal score of 2. Subsequently, the mice recovered, and by day 13 p.i., these mice no longer showed disease symptoms (**Figure 4D**, gray line). In mice treated with rapamycin, disease symptoms also reached a maximal score of 2 at day 8 p.i., but this score persisted until day 11 p.i. Thereafter, the mice began to recover slowly, but only on day 17 p.i. they showed no more symptoms (**Figure 4D**, orange line). Transfer of Tregs also resulted in an increase in DSS and a maximum score of 2 at day 10 p.i. Disease symptoms persisted until day 17 p.i. (**Figure 4D**, purple line). Compared to control animals, both treatment with rapamycin and transfer of Tregs resulted in a prolongation of disease duration. The results suggest that CD4^+^/CD25^+^FoxP3^+^ Tregs have an impact on pathogenesis by influenza viruses.

### MEK-inhibitor treatment reduces the induction of regulatory T cells *in vitro* and *in vivo*

**Figure 5:**
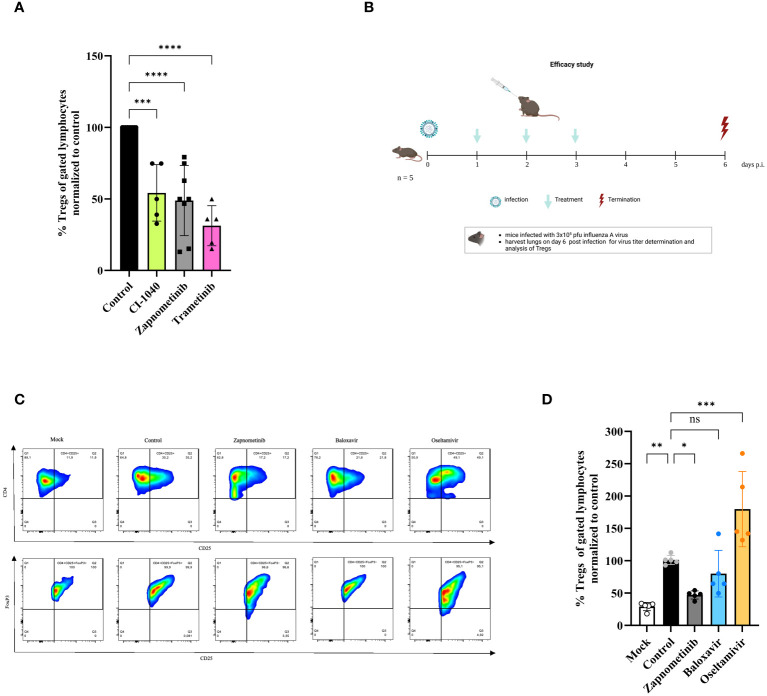
Effect of different therapeutics on the induction of regulatory T cells *in vitro* and *in vivo.* Studies on the effect of different drugs on Treg response. **(A)** *In vitro* study on Tregs. Human Tregs were isolated from human PBMCs of healthy volunteers, sorted from pre-enriched CD4^+^ cells and *in vitro* stimulated for 36 h with anti-CD3/CD28 coated MACSiBead in the presence or absence of class one MEK-inhibitors, followed by Foxp3 staining and flow cytometry analysis. The data are shown as %Tregs (of gated lymphocytes) normalized to untreated control and presented as mean ± SD (n = 5–8). One-way ANOVA with Sidak correction was used for statistical analysis. p = 0.0001 is represented with *** and p< 0.0001 with ****. **(B-D)** Mice were infected with 3 x 10^5^ pfu influenza A virus and treated twice daily with either zapnometinib, baloxavir, oseltamivir or solvent control for 3 days and sacrificed 6 days p.i. Lungs were harvested on day 6 p.i. and analyzed for the presence of Tregs. **(B)** Schematic of the *in vivo* efficacy study. Clinical signs were assessed once daily and included body weight and disease symptom score. This figure was created with BioRender.com. **(C)** Gating strategy for the quantification of Tregs in the lung of IAV infected and treated mice. CD4^+^ cells were gated for CD4^+^CD25^+^ cells and further for FoxP3^+^. **(D)** *In vivo* effect of zapnometinib, baloxavir and oseltamivir on the induction of Tregs after infection with IAV compared to control. Data presented as mean ± SD (n = 5). One-way ANOVA with Sidak correction was used for statistical analysis (*p< 0.05, **p< 0.005, ***p< 0.001, ns, not significant).

Based on these experiments one might argue that CD4^+^/CD25^+^FoxP3^+^ Tregs are involved in the pathogenicity and disease severity in IAV infected mice. We have previously reported that MEK-inhibition has an influence on the disease severity after IAV infection in mice (30). Thus, the influence of MEK-inhibition on CD4^+^/CD25^+^FoxP3^+^ Tregs was investigated in mice after IAV infection and in human PBMCs. CD4^+^/CD25^+^FoxP3^+^ regulatory T cells were isolated from human blood of healthy volunteers and stimulated in the presence or absence of the MEK-inhibitors zapnometinib, CI-1040 and trametinib for 36 h, followed by the quantification of CD4^+^/CD25^+^FoxP3^+^ Tregs via FACS analysis. **Figure 5A** shows that the formation of Tregs was significantly blocked in the presence of MEK-inhibitors. Treatment with CI-1040 reduced the amount of Tregs by 45.80 ± 19.75% compared to the untreated control. In cells treated with zapnometinib the amount of Tregs was decreased by 51.10 ± 24.55% and trametinib treatment reduced the amount of Tregs by 68.75 ± 14.06% (**Figure 5A**). These results demonstrate that MEK-inhibition reduces the formation of Tregs.

Next, the efficacy of zapnometinib to prevent the induction of Tregs was investigated in the IAV mouse model and compared with standard of care treatment for infection with IAV. Therefore, mice were infected with high dose (3 x 10^5^ pfu) of IAV and 24 hours later treated twice daily with 25 mg/kg zapnometinib, 10 mg/kg oseltamivir or 15 mg/kg baloxavir marboxil for 3 days. The mice were sacrificed on day 6 p.i., and the lungs were examined for the presence of Tregs. The study design is summarized in **Figure 5B** and a part of the gating in **Figure 5C**. IAV infection induced a significant higher amount of Tregs compared to non-infected Mock control (29.15 ± 6.39%, white bar, **Figure 5D**). Treatment of mice with zapnometinib significantly reduced the induction of Tregs (by 53.02 ± 6.27%, grey bar) compared to control (black bar). In contrast, baloxavir (19.98 ± 36.09%, blue bar) had no effect on Treg induction, whereas oseltamivir resulted in an increase of Tregs (to 179.77 ± 58.15%, yellow bar). Thus, MEK-inhibitor treatment reduces CD4^+^/CD25^+^FoxP3^+^ Tregs from human PBMCs and in an IAV mouse model.

## Discussion

In recent years, there has been an increasing number of studies on CD4^+^/CD25^+^FoxP3^+^ regulatory T cells (Tregs) and their involvement in viral infections. While some studies have demonstrated that Tregs are necessary to protect the host from various diseases, others have shown that CD4^+^/CD25^+^FoxP3^+^ Tregs may be responsible for the severity of the disease (19–22). However, the role of Tregs in acute infections, such as influenza A virus (IAV) infection, is poorly understood.

In the present study, we demonstrated that CD4^+^/CD25^+^FoxP3^+^ Tregs play an important role in IAV-induced pathogenesis in mice. When comparing mild and severe influenza mouse model, we could show that the induced Tregs in the lungs of mice that developed severe influenza disease symptoms spread before the expansion of effector T cells. However, the viral load was not decisive of early Treg expansion. There was no quantitative difference in infectious viral particles in the lungs after low or high infection doses on day 3 p.i. and day 9 p.i (**Figure 1E**). This is in line with influenza studies where no correlation between viral load and the severity of the disease could be established (36). Severe influenza disease is associated with T cell deficiency and aberrant responses (37, 38). We observed no quantitative difference in the number of CD4^+^ and CD8^+^ T cells between low and high dose infection, but a significant shift from a Th1 to a Th2 response and a lower amount of CD8^+^ effector T cells after high dose IAV infection (**Figures 3C, G**). We hypothesize that one reason for the impaired altered immune response during severe pathogenesis is the very rapid expansion of CD4^+^/CD25^+^/FoxP3^+^ Tregs in the lung. This hypothesis is further supported by higher levels of CD4^+^/CD25^+^/FoxP3^+^ Tregs in mice with severe disease, indicating that CD4^+^/CD25^+^/FoxP3^+^ Tregs might reduce virus-specific CD8^+^ T cell effector function, which has also been demonstrated for hepatitis C virus infections (39). A shift from a CD4^+^ Th1 to a CD4^+^ Th2 response can also be attributed to Tregs and has been confirmed in other infectious diseases (18, 40). A single transfer of Tregs from IAV infected mice that developed severe disease exacerbated and prolonged the course of pathogenesis in mice infected with a low dose of IAV (**Figure 4**). This demonstrates the pathogenic potential of CD4^+^/CD25^+^/FoxP3^+^ Tregs. However, our investigations cannot distinguish functional differences of Tregs following higher infection dose compared to Tregs following lower infection dose. During low dose infection, induction of Tregs occurs after expansion of effector T cells.

Since Tregs can inhibit innate immune cells during IAV infection, it could also be argued that the high number of Tregs during severe influenza leads to inhibition of the innate immune response in addition to inhibition of the adaptive immune response (41). The present study cannot confirm this observation since there were no differences in the amount of Natural Killer (NK) cells and Natural Killer T cells (NKT) cells after low and high dose infection (**Supplementary Figure 1**). Although Treg expansion in the lungs of mice developing mild influenza symptoms peaked at the same level as in mice developing severe influenza symptoms (**Figures 1C**, **2E**), Treg expansion here is detectable only after induction of effector CD8^+^ T cells to control the cellular immune response to the virus. These results suggest that early induction of Tregs alter the function of virus specific CD8^+^ T cells. These findings were also supported by different virus infection models, were Tregs suppressed the function of activated CD8^+^ T cells (19, 39, 42). Furthermore, in tuberculosis studies, Tregs have been shown to delay effector T cells in the lung during the early phase of infection (43). In contrast, in a study with mouse hepatitis virus, virus-specific Tregs suppress the pathogenic effector T cell response and thereby attenuate the disease.

Studies suggest that infectious and inflammatory disease treatment will largely benefit from manipulation of these Tregs (44). Since Tregs limit the effector immune response, it is of great importance to study the effects of inhibiting the activation of Tregs. In a study with the herpes simplex virus (HSV), depletion of Tregs in mice resulted in an increase of the immune response, and viral clearance (18, 45). Thus, we raised the question of whether the induction of Tregs can be prevented. Since it is known that Treg activation upon antigen stimulation depends on the Raf/MEK/ERK signaling pathway and, moreover, that IAV replication is strongly dependent on this signaling pathway, our study aimed to investigate the influence of MEK-inhibition on the induction and activation of CD4^+^/CD25^+^/FoxP3^+^ Tregs. We could show that MEK-inhibitors significantly reduce the induction of Tregs *in vitro* as well as *in vivo*. Our results confirm the findings from a study with tuberculosis and HIV patients in which MEK-inhibitors prevent the activation of Tregs in human PBMCs (24). In a mouse model, we were able to demonstrate that the standard influenza drugs baloxavir and oseltamivir effectively reduced viral load. But, while baloxavir had no impact on the reduction of regulatory T cells oseltamivir treatment even increased the amount of Tregs (**Figure 5D**). The MEK-inhibitor zapnometinib reduced the amount of CD4^+^/CD25^+^/FoxP3^+^ Tregs. Additionally, MEK-inhibition with zapnometinib resulted in reduced viral load (30, 46). Independently of individual therapy, the combined use of zapnometinib with direct-acting antiviral drugs (DAAs) is also conceivable. Some colleagues report a synergistic enhancement of the effects of these drugs (33, 47). For instance, it has been demonstrated that zapnometinib, when combined with baloxavir, enhances therapeutic efficacy *in vitro* and exerts a dose-dependent inhibitory effect on IAV replication across various strains and subtypes (33). Furthermore, a synergistic effect on viral replication was observed in SARS-CoV-2 infection through the combined use of zapnometinib with molnupiravir, remdesivir, as well as with nirmatrelvir and ritonavir *in vitro* (47).

In addition to the inhibition of the MEK pathway, the suppression of p38 kinase demonstrated antiviral efficacy and a concurrent reduction in pro-inflammatory cytokines during both influenza virus and SARS-CoV-2 infections (48, 49). The functional role of p38 in regulatory T cells (Tregs) appears to be nuanced, exhibiting a dual nature, with effects contingent upon the specific context of the immune response. The absence of both p38α and p38β isoforms in murine models resulted in lymphoid atrophy and an augmented frequency of regulatory T cells. Furthermore, pharmacological inhibition of p38 replicated similar outcomes (50).

Th1 T cells are essential for the host defense toward intracellular pathogens. In our study, we demonstrated that the low IAV infection dose tended to result in a sufficient Th1 immune response to clear the viral infection and we observed a trend towards a Th2 immune response after a high dose of infection. These results are consistent with data from previous studies, which have already shown that low-dose infection or low antigen concentration favors the induction of Th1 responses, whereas high-dose infection preferentially induces Th2 responses (51–53). Our findings are consistent with data from a severe acute respiratory syndrome coronavirus 2 (SARS-CoV-2) study in which COVID-19 patients exhibited an overly reactive Th2 response to the virus (54). Therefore, we conclude that the type of Th response has been shown to be critical for disease resolution. It was already shown that with the MEK-inhibitor treatment modulates the immune response and shift it towards a Th1 response (55).

The regeneration of the epithelial barrier is crucial following respiratory viral infections to preserve lung function and mitigate the risk of secondary infections. A subset of Treg cells in the lungs play a pivotal role in maintaining blood oxygenation post-influenza virus infection, primarily by producing the epidermal growth factor receptor (EGFR) ligand, amphiregulin (AREG). However, the precise mechanisms by which Treg cells interact with progenitors in the alveolar niche remain elusive (56, 57). In a separate study, Xie et al. (58) observed elevated expression of AREG in lung tissues of mice infected with SARS-CoV-2 compared to mock-infected tissues. This finding raises the intriguing possibility that the increased AREG expression may contribute to the activation of MEK1/2 in lung tissues following SARS-CoV-2 infection. Together, these studies shed light on the intricate relationship between Treg cells, AREG expression, and the activation of MEK1/2 in the context of respiratory viral infections. While the exact mechanisms by which Treg cells engage with alveolar progenitors warrant further investigation, the observed elevation of AREG in response to viral infections, such as influenza and SARS-CoV-2, suggests a potential link between Treg-mediated regeneration and MEK1/2 activation in the lung tissues. A limitation of the present study is that the influence of MEK-inhibition on these pro-repair Tregs could not be investigated.

One limitation of our study is that the small number of animals increases the risk of not detecting all effects that might occur with a larger sample size. A study of the effects of zapnometinib on effector T cells and cytokine reduction would further strengthen our results but could not be investigated in the scope of this study. However, since the effect of zapnometinib on cytokine expression has already been confirmed (31), and the effect of trametinib on effector T cells has been shown, we therefore speculate on a class effect for zapnometinib that might be confirmed with further studies. In addition, it might be useful to perform histologic staining to evaluate the effect of zapnometinib treatment on alveolar regeneration. Unfortunately, we were not able to do this in the study we conducted. However, histopathologic examinations were performed in a study of SARS-CoV-2 infected hamsters (http://dx.doi.org/10.2139/ssrn.4645186). It was found that treatment with zapnometinib significantly reduced the percentage of lung lesions in the lung tissue compared to the vehicle-treated group, indicating a beneficial effect of zapnometinib treatment. In addition, inflammation, as measured by the number of inflammatory cells infiltrating the tissue, was less severe in the zapnometinib-treated groups than in the vehicle-treated control group. Zapnometinib-treated hamsters showed less alveolitis with fewer cellular inflammatory infiltrates containing few macrophages and neutrophils, and no hemorrhage, edema, or type II pneumocyte hyperplasia. Zapnometinib treatment probably has a similar effect in influenza-infected mice. Since MEK inhibitors interfere with a general cellular metabolic pathway, there is a suspicion that this is associated with risks. We were able to demonstrate in a Phase 2 study in hospitalized COVID-19 patients that a six-day administration of zapnometinib was well-tolerated (59).

In conclusion, we were able to demonstrate the influence of Tregs on the pathogenesis of IAV infection. We could show that Tregs are significantly involved in the progression of the disease by modulating the immune response. Furthermore, we were able to show that the induction of Tregs is reduced by treatment with MEK-inhibitors. This study provides important data for the dual effect of MEK-inhibitors. Besides the reduction of viral load, regulatory T cells could be reduced, and an effector immune response supported. This could mitigate the progression of influenza and be beneficial in other viral infections as well.

## Data availability statement

The original contributions presented in the study are included in the article/**Supplementary Material**. Further inquiries can be directed to the corresponding author.

## Ethics statement

The studies involving humans were approved by Ethics Board of the Medical Faculty of the Eberhard Karls University of Tübingen and the University Hospital Tübingen. The studies were conducted in accordance with the local legislation and institutional requirements. The participants provided their written informed consent to participate in this study. The animal study was approved by Regional Council Tuebingen (IM 01/21 G). The study was conducted in accordance with the local legislation and institutional requirements.

## Author contributions

JK: Conceptualization, Data curation, Investigation, Methodology, Writing – original draft, Writing – review & editing. AV: Data curation, Methodology, Writing – review & editing. YF: Data curation, Methodology, Writing – review & editing. ME: Data curation, Writing – review & editing. AS: Data curation, Methodology, Writing – review & editing. RZ: Data curation, Methodology, Writing – review & editing. OP: Conceptualization, Resources, Supervision, Writing – review & editing.
