# Supplementary material for: MEK-inhibitor treatment reduces the induction of regulatory T cells in mice after influenza A virus infection
Source: Front Immunol. 2024 Jun 24;15:1360698. doi: 10.3389/fimmu.2024.1360698 (PMC11228811; doi:10.3389/fimmu.2024.1360698)
Supplement: Supplementary file 1 [file DataSheet_1.pdf]

Supplementary Material

Supplementary Figure

We also quantified the cells of the innate immune response in the lungs of mice after infection with the low or high dose of IAV. CD11c+ dendritic cells (DC) were significantly increased on day 1 p.i. in the lungs of mice infected with either the low (10.12 ± 1.53%) or a high virus dose (9.86 ± 2.49%) compared to uninfected controls (5.18 ± 0.64%, Supplement Figure 1A, grey dashed line). Subsequently, the percentage of CD11c+ DCs decreased and on day 6 p.i. 2.88 ± 2.01% CD11c+ DCs were still detected with the low dose whereas the high infection dose resulted in a significant reduction to 1.25 ± 0.19% CD11c+ DCs (Supplement Figure 1A). CD11b+ CD11c- macrophages were also significantly increased on day 1 p.i., at both the low (5.01 ± 1.04%) and high dose (9.21 ± 1.07%) compared to uninfected control (2.47 ± 0.57%, Supplement Figure 1B, grey dashed line). In contrast, significantly more CD11b+ CD11c- macrophages migrated into the lungs with the high dose IAV infection. On day 3 p.i., the proportion was comparable between the two infection doses at about 6%, and at day 6 p.i. the percentage further decreased to 4.17 ± 0.61% with the low dose and to 3.19 ± 1.07% with the high dose infection (Supplement Figure 1B). Ly6G+ neutrophils were significantly increased compared to controls (10.01 ± 1.86%, Supplement Figure 1C, grey dashed line) only at day 3 p.i. (low dose: 28.46 ± 3.37%, high dose: 39.44 ± 11.41%) and day 6 p.i. (low dose: 39.40 ± 17.92%, high dose: 49.98 ± 5.05%); no significant differences were found between the low and high infection doses (Supplement Figure 1C). Natural killer (NK) cells were significantly increased at day 3 p.i. in mice infected with the high dose (20.72 ± 5.98%) compared to control (4.07 ± 1.16%). High-dose infection caused a significantly higher increase in NK cells at day 3 p.i. compared to low-dose infection (12.27 ± 1.38%). On day 6 p.i. both infection doses led to a significant increase compared to control (low dose: 14.96 ± 6.67%, high dose: 20.90 ± 4.62%) (Supplement Figure 1D). Also, the amount of Natural Killer T -cells (NKT) was significantly increased in the lungs of mice infected with the high dose (0.51 ± 0.11%) compared to uninfected controls (0.14 ± 0.04%) on day 3 p.i. At day 6 p.i., the number of NKT cells was significantly increased compared to the uninfected control after both low dose (0.66 ± 0.42%) and high dose (0.87 ± 0.21%) infection (Supplement Figure 1E).

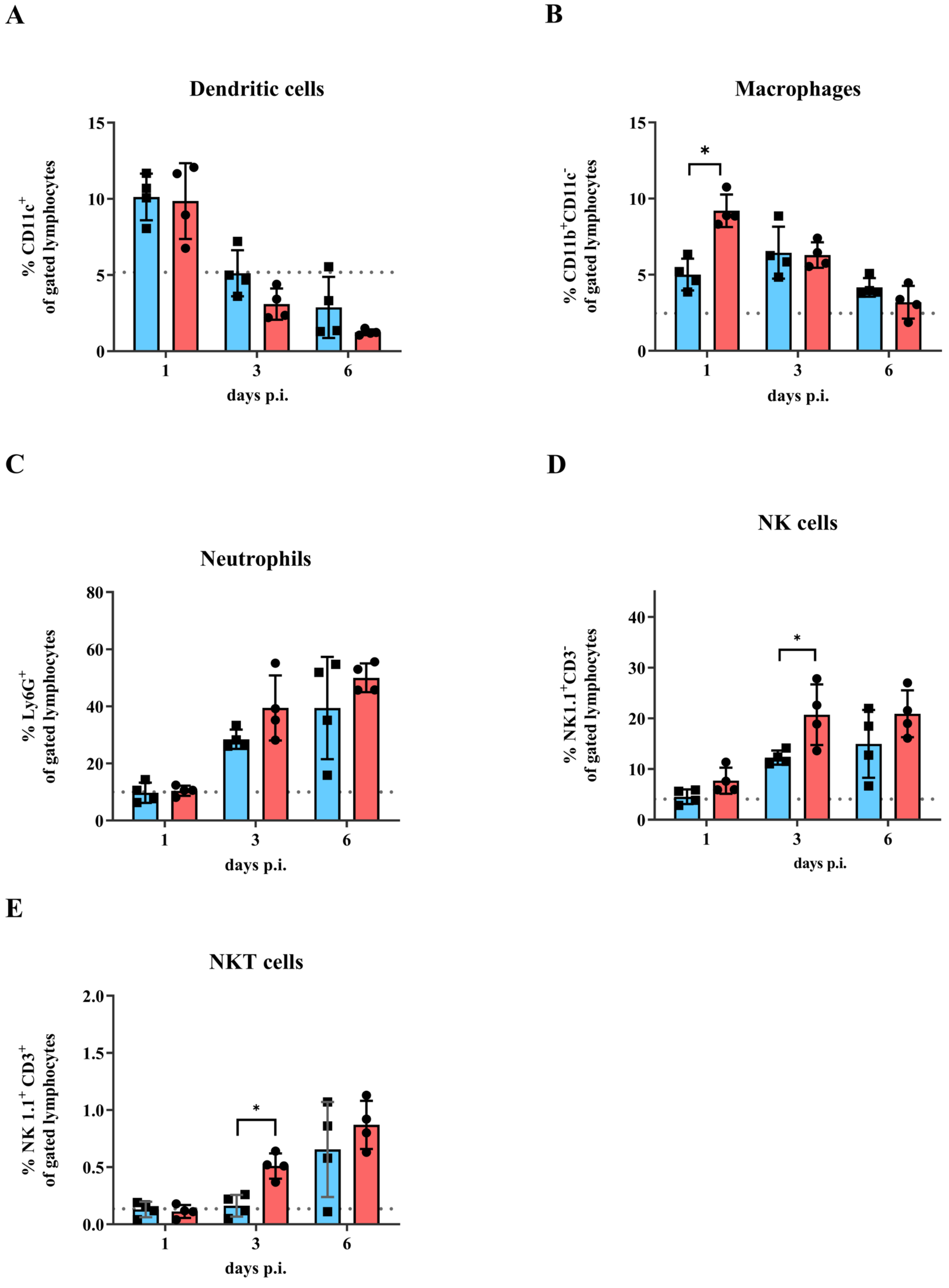

Supplement Figure 1. Numbers of innate immune cells differ in mice infected with low or high infection dose of influenza A virus at early timepoints of disease. Mice were infected with either high dose (red bars) or low dose (blue bars) of influenza A virus and the percentage of (A) dendritic cells, (B) macrophages, (C) neutrophils, (D) NK cells or (E) NKT cells of gated lymphocytes were analyzed at 1-, 3-, and 6-days p.i by flow cytometry compared to uninfected control (dashed grey line). Data presented as mean ± SD (n = 4). Comparison between the two groups low dose (blue bars) and high dose (red bars) at the same time point is indicated by the black stars and was analyzed with two-way ANOVA r (black *with bracket, $p < 0.05$).

Supplementary Tables

Supplementary Table S1. Score and specific characteristics for the assessment of health status of the survival study.

Score	Specific characteristics
0	healthy mouse
1	ruffled fur
2	ruffled fur, breathing sounds, slight body curvature
3	ruffled fur, breathing sounds, body curvature, lethargy
4	dead mouse

Supplementary Table S2. Mean disease symptoms score of the survival study.

Days p.i.	Low dose	High dose
0	0	0
1	0	0
2	0	0.33
3	0.33	1.33
4	0.67	1.33
5	0.67	2
6	1.33	2.33
7	1.67	2.67
8	2	2.67
9	1.67	3
10	0.67	3.5
11	0.67	3
12	0.67	4
13	0	4
14	0	4
15	0	4
16	0	4
17	0	4
18	0	4
19	0	4
20	0	4

Mice were infected with either the high dose ($3x10^5$ pfu) or low dose ($5x10^3$ pfu) of IAV and observed for 19 days. Disease status was observed. A healthy animal received a value of "0" while a dead animal received a maximum value of "4". Values are delineated as mean (n=3).

Supplementary Table S3. Categories, symptoms, and score for the assessment of health status of the efficacy study.

Category	Symptoms	Score
Breathing	Inconspicuous	0
	slightly aggravated	1
	moderately aggravated curvature	2
	clearly aggravated/strong breath sounds	3
Environment	inconspicuous	0
	no contact to other animals	1
	crouches reinforced in one place	2
	shows apathetic behavior	3
Body posture	inconspicuous	0
	small kyphosis	1
	moderately kyphosis	2
	severe kyphosis	3
Fur care	inconspicuous	0
	slightly ruffled fur	1
	moderately ruffled fur/slightly dirty	2
	strong ruffled fur/dirty	3

Infected mice were observed daily and the DSS was determined using these categories. In this study, termination criteria were as soon as a mouse received a score of 3 in one category or a total score of >7.

Supplementary Table S4. Mean disease symptoms score of the efficacy study.

Days p.i.	Control	Zapnometinib	Baloxavir	Oseltamivir
0	0	0	0	0
1	0	0	0	0
2	0.18	0.23	0	0.80
3	0.09	0.06	0.06	0.20
4	1.34	0.79	1.06	1.20
5	1.48	1.90	1.46	0.40
6	2.38	1.79	1.46	2.00

Mice were infected with the high dose ($3x10^5$ pfu) of IAV and observed for 7 days. Disease status was observed. A healthy animal received a value of "0" while a dead animal received a maximum value of "7". Values are delineated as mean (n=5).
